# Antimicrobial-Induced Cytopenia and Bone Marrow Hypocellularity in Patients with Cirrhosis

**DOI:** 10.1155/2018/4029648

**Published:** 2018-05-14

**Authors:** Anupama Patil, Vikas Khillan, Monika Thakur, Pratibha Kale, Chhagan Bihari

**Affiliations:** ^1^Department of Clinical Hematology, Institute of Liver & Biliary Sciences, New Delhi, India; ^2^Department of Microbiology, Institute of Liver & Biliary Sciences, New Delhi, India

## Abstract

There is great variation in cytopenias in cirrhotic patients with same severity and hypersplenism and their causative factors are not clear. Recent studies have highlighted the role of gut microbiome in regulation of constant and emergency hematopoiesis. Broad-spectrum antibiotics can disrupt the homeostatic or adaptive microbiota in cirrhosis, leading to impaired hematopoiesis and a higher susceptibility to infections. We studied all patients with cirrhosis with cytopenia (anemia, leucopenia, and/or thrombocytopenia), admitted in the Institute of Liver & Biliary Sciences, between January 2016 and July 2017, who underwent a bone marrow examination. The effect of the different antimicrobial agents on peripheral blood counts and bone marrow cellularity was assessed. A total of 196 patients' data was analyzed for this study. Patients on antimicrobials (*n* = 115) had significantly lower hemoglobin (*p* < 0.001), total leucocyte count (*p* = 0.048), and platelet count (*p* = 0.043) compared to patients not on antimicrobials. On unadjusted analysis, significant association with thrombocytopenia existed in beta-lactams (OR = 1.56, 95% CI = 1.06–2.40), quinolones (OR = 1.66, 95% CI = 1.11–2.61), and antifungals (OR = 2.24, 95% CI = 1.96–4.34). Cephalosporins were found to be significantly associated with anemia (OR = 1.91, 95% CI = 1.07–3.41). Patients who received antimicrobials had hypocellular marrow (*p* < 0.001) as compared to nonrecipients of antibiotics. The adjusted analysis showed that quinolones and beta-lactam antibiotics are the drug classes having significant association with thrombocytopenia and alternative class of drug should be explored in these patients to avoid severe thrombocytopenia.

## 1. Introduction

Patients of chronic liver disease (CLD) and end-stage cirrhosis have varying grades of cytopenia and other hematologic derangements either due to the compromised synthetic capacity of the diseased liver itself or resulting from the innumerable medical or surgical insults [1]. These patients experience complications related to uni- or multilineage peripheral cytopenias. It is known that patients with one or more cytopenias have prolonged hospitalization and reduced survival rates [2, 3]. However, the grades of cytopenias in patients with same severity of CLD and their causative factors are not clear. It is believed that such cytopenias are caused by hypersplenism but extent of cytopenias caused by hypersplenism is variable. It is possible that there may be interplay of numerous other factors that cause cytopenias in these patients.

Several recent studies have elucidated the role of gut microbial dysregulation attributed to antimicrobial therapy [4–7]. Infections are very common in advanced cirrhosis and antimicrobial prophylaxis is necessary in patients with cirrhosis who have gastrointestinal bleeding, as well as in invasive procedures such as transjugular intrahepatic portosystemic shunt [8, 9]. Hence a decreased gut microbial diversity is especially relevant in patients with CLD as majority of them are treated with antimicrobials. Most broad-spectrum antibiotics impact not only the harmful bacteria but also healthy ones. Apart from impairment of the healthy gut microbiota, antibiotics have a direct harmful effect on the intestinal epithelia and facilitate spread of antibiotic-resistant microorganisms too [10]. Owing to the close relationship between gut and liver via the “gut-liver” axis, gut microbiota plays a major role in the pathogenesis of many CLDs, as suggested by increasing evidence [11–14].

Recent studies have also highlighted the role of gut microbiota in regulation of hematopoiesis [4, 15]. The gut microbial products such as lipopolysaccharides stimulate lymphocytes, macrophages, and dendritic cells in the lamina propria which, in turn, produce a series of extrinsic stimuli. All these microbial and cellular stimuli work together to sustain steady-state hematopoiesis and also induce emergency myelopoiesis [6, 16]. Treatment with broad-spectrum antibiotics can disrupt the balance and diversity of gut microbiota, leading to impaired hematopoiesis and a higher susceptibility to infections [7]. Hence, it is possible that cytopenias in patients with CLDs, in part, could be attributed to fecal dysbiosis induced by antimicrobial therapy.

There is a paucity of reports that specifically address antimicrobial-induced cytopenias in the CLD population. Therefore, we analyzed cases of cirrhosis on antimicrobials that developed uni- or multilineage cytopenias in our hospital over a 1-year period to examine the spectrum of the implicated agents. We also examined the bone marrow aspiration (BMA) and/or biopsy (BBx) findings to determine the histological diagnoses.

## 2. Patients and Methods

### 2.1. Patient Population

We studied all patients with cirrhosis with cytopenia (anemia, leucopenia, and/or thrombocytopenia), admitted in the Institute of Liver & Biliary Sciences, between January 2016 and July 2017, who underwent a bone marrow (BM) aspiration and/or biopsy procedure. Cirrhosis was diagnosed based on clinical, laboratory, and radiological evidence. The etiological categories of CLD included alcoholic and nonalcoholic fatty liver disease (NAFLD) and viral, cryptogenic, and vascular disorders of the liver. Exclusion criteria were patients with hematological/hepatic malignancy and cases with missing records.

### 2.2. Clinical and Laboratory Data

The clinical diagnosis and etiology of CLD was noted from the patient case record forms. Venous blood samples were routinely collected from all patients to assess complete blood counts, prothrombin time/international normalized ratio (PT/INR), liver function tests, and serum urea and creatinine levels. The hematological counts were assayed using EDTA-anticoagulated blood on LH750 (Beckman Coulter Inc., Brea, California, USA) and Horiba ABX Pentra DX 120 (Horiba Medical, Montpellier, France). Citrated blood sample (with sodium citrate as anticoagulant in a ratio of 9 : 1) was used for studying the coagulation tests performed on fully automated coagulometer (Sysmex CA 1500; Sysmex Corporation, Kobe, Japan). The biochemical tests were performed on DXC 600 Pro (Beckman Coulter Inc., Brea, California). Serum procalcitonin levels were measured using the Maglumi 1000 Analyzer chemiluminescence immunoassay system (SNIBE Co Ltd, Guangdong, China). Serum ferritin and C-reactive protein (CRP) levels were analyzed by nephelometry (Dade Behring BN ProSpec; Siemens Healthcare Diagnostics, Marburg, Germany). TNF-*α* levels were determined using the automated Immulite CLIA system (Siemens, Germany).

The effect of the different antimicrobial agents on peripheral blood counts in patients with CLD was explored. Cytopenias were defined as follows: hemoglobin (Hb) < 9 g/dL, total leucocyte count (TLC) < 4,000/*μ*L, and platelet count (Plt) < 100,000/*μ*L [17]. Details regarding number and duration of antimicrobials given to each patient were recorded. Model for End-Stage Liver Disease (MELD) scores was used to assess the severity of the CLD. The ultimate outcome of each patient, whether discharged/expired/left against medical advice (LAMA), was also noted.

### 2.3. Statistical Analyses

Data in Excel (Microsoft, Redmond, WA, USA) sheet were imported into and analyzed using STATA (version 12.1 STATA Corp., College Station, TX, USA). To begin baseline characteristics were summarized using frequency, proportion, mean (SD), and median (IQR). The difference of distribution (unadjusted analysis) between numeric variables among groups was analyzed using the *t*-test or one-way analysis of variance (ANOVA). Mann–Whitney's or Kruskal-Wallis test was used for nonparametric data. Logistic regression was used to estimate the ORs and 95% CIs for the association between exposure to the medication classes and cytopenias.

## 3. Results

### 3.1. Patient Characteristics

A total of 196 patients' data was analyzed for this study.  Table 1 displays the general characteristics of the patients with regard to age, gender, clinical diagnosis, antimicrobials given, MELD scores, and outcome (death/discharge/LAMA). The mean age of these patients was 46 ± 15 years, with 157 (80%) males. The etiologies included ethanol (31.6%), nonalcoholic steatohepatitis (NASH) (25.4%), cryptogenic (23%) and hepatotropic viruses (4.6%), vascular pathology (10.2%), and others (5%). Of the total 196 patients, 115 were given antimicrobials as part of their treatment whereas the remaining 81 received none. As evident from Table 1, there is no statistically significant difference in the MELD scores and mean spleen size between the two groups of patients, making them comparable as per severity of liver disease and presence of hypersplenism.

### 3.2. Use of Antimicrobial Agents

The antibiotics most commonly used were 3rd-generation cephalosporins (cefotaxime, ceftriaxone, cefixime, and ceftazidime), carbapenems (meropenem, doripenem, ertapenem, and imipenem), beta-lactams (piperacillin/tazobactam, sulbactam, and co-amoxiclav), quinolones (ciprofloxacin, norfloxacin, and levofloxacin), and antifungals (fluconazole). The median length of an antibiotic course was 7 days (IQR 5–9).

### 3.3. Range of Cytopenias

As evident from Table 1, there was significant difference in the hemoglobin (*p* < 0.001), total leucocyte count (*p* = 0.048), and platelet count (*p* = 0.043) between the two groups of patients.  Table 2 depicts the range of cytopenias and outcomes in different categories of antimicrobials in patients with CLD. Among patients who were given antimicrobials (*n* = 115), only 28 (24.3%) patients had a normal blood cell count. The remaining 87 had peripheral cytopenias, in which unilineage cytopenias accounted for 21.7% (25/115), bilineage cytopenias accounted for 28.7% (33/115), and trilineage cytopenias accounted for 21.7% (25/115). As shown in Table 1, patients on antimicrobial therapy had significantly lower values of hemoglobin (*p* < 0.001), total leucocyte count (*p* = 0.048), and platelet count (*p* = 0.043) when compared to those not on any antimicrobials. In the unadjusted analysis, drug classes with a statistically significant association with thrombocytopenia were beta-lactams (OR = 1.56, 95% CI = 1.06–2.40), quinolones (OR = 1.66, 95% CI = 1.11–2.61), and antifungals (OR = 2.24, 95% CI = 1.96–4.34). Further, cephalosporins were found to be significantly associated with anemia (OR = 1.91, 95% CI = 1.07–3.41). We found no statistically significant association between any particular antimicrobial agents and leucopenia.

After adjusting for MELD score, spleen size, and procalcitonin, beta-lactam antibiotics (OR = 1.62, 95% CI = 1.64–2.96) and quinolones (OR = 1.53, 95% CI = 1.47–2.51) were the only drug classes with a statistically significant association with thrombocytopenia (Table 3). Antifungals were no longer statistically associated with thrombocytopenia after adjustment (OR = 1.64, 95% CI = 0.86–4.36). Likewise, cephalosporins too lacked significant association with anemia (OR = 1.62, 95% CI = 0.97–3.41).

We found no statistically significant association between carbapenems and other antimicrobials and cytopenias.

### 3.4. Bone Marrow Findings

Clinical indications for BMA/BBx were to investigate cytopenias and to rule out infections/hematological neoplasms/hemophagocytic lymphohistiocytosis (HLH). In both the groups, majority of patients had a reactive marrow and twenty-five (21.7%) patients who received antimicrobials had hypocellular marrow. In contrast none of the patients in the nonantimicrobial group had marrow hypocellularity (Table 1). In patients with hypocellular marrow (*n* = 25) there was no significant difference among the five classes of antimicrobials studied (cephalosporins *p* = 0.776, carbapenems *p* = 0.063, beta-lactams *p* = 0.677, quinolones *p* = 0.770, and antifungals *p* = 0.469).

There was no significant difference between the two groups with regard to marrow histology (myeloid : erythroid ratio, marrow fibrosis, neovascularization, CD34 percentage, etc.)

### 3.5. Outcome

Of the 196 patients, 169 (86.2%) survived and were discharged, 13 (6.6%) died, and the remaining 14 (7.1%) patients left against medical advice. There was no statistically significant difference in the outcomes of the patients in the two groups [Table 1].

## 4. Discussion

To our knowledge, this is the first study to evaluate the risk of cytopenias associated with the use of commonly prescribed antimicrobial agents in the specific cohort of patients with CLD. The adjusted analysis showed that quinolones and beta-lactam antibiotics were the only drug classes with a statistically significant association with thrombocytopenia. Further, we found no association between cephalosporins, carbapenems, antifungals, and peripheral blood cytopenias.

Our findings are in line with previous studies (observing a generalized population of sick patients) which have highlighted cases of thrombocytopenia associated with the use of quinolones [18–20]. It has been postulated that the mechanism of this association is due to drug-dependent, platelet-reactive antibodies causing complement-mediated destruction of platelets [20]. Further, recent reviews have suggested that this immune thrombocytopenia of quinolones may be explained by the structural relationship between quinolones and quinine, which is well known to induce platelet-reactive antibodies [21]. Researchers have also shown evidence for an increased risk of thrombocytopenia with use of beta-lactam antibiotic agents [22–24]. In these cases, too, the underlying mechanism was thought to be covalent binding of drug-dependent antibodies to platelet membrane resulting in platelet destruction. We found no association between other agents such as cephalosporins, carbapenems, and antifungals and cytopenias in our patients.

The strengths of our study include the patient population selected for the analysis and the robust multivariate analysis implemented for adjusting for the various confounding factors. However, our study had a few limitations. For instance, we could not evaluate the potential effect of different drug combinations on cytopenias. The analysis of the various antimicrobial agents as groups could have masked effects of individual drugs. Also, we analyzed only the most commonly used medications in our study population and not every antimicrobial agent that has been known to be associated with cytopenias.

In patients with CLD, cytopenias were considered to be caused mostly due to hypersplenism [25]. But this hypothesis has never been proven beyond any doubt. It is known that anemia due to excessive splenic pooling or phagocytosis is rare [26]. Also, neutropenia does not always resolve after splenic embolization or splenectomy [27]. With regard to thrombocytopenia, studies have failed to demonstrate any direct correlation between platelet count and spleen size [28]. In recent years, several other factors have also been known to contribute to the decreased blood counts in these patients, namely, toxic effects of hepatic virus/alcohol [29, 30], hypofunctioning of the diseased liver [31, 32], and gastrointestinal bleeding [26]. An interesting revelation in cirrhosis in recent years is the occurrence of fecal dysbiosis in patients with cirrhosis. Most of the precirrhotic liver diseases and cirrhosis are associated with intestinal bacterial overgrowth and also increased gut wall permeability. This has been described as the leaky gut hypothesis. Changes in the gut microbiota also lead to the enhanced release of proinflammatory cytokines which further enhance translocation and in turn links to development of many complications of CLD, as well as progression of disease. As is already well known, antimicrobials, especially those with broad-spectrum action, impact not only harmful bacteria, but also healthy ones [7]. This is of particular relevance in patients with CLD; antibiotics are considered a cornerstone in the management of CLD and its various complications. It is interesting to note here the role of microbial metabolites on hematopoiesis. Josefsdottir et al. have shown that commensal gut microbes regulate and sustain normal steady-state hematopoiesis [33]. They further demonstrate that broad-spectrum antibiotic treatment of mice for >2 weeks depletes the gut microbial flora, which ultimately leads to a decrease in the numbers of hematopoietic progenitors in the bone marrow and concomitant cytopenias. This is attributed not to the toxic effect of antibiotics on hematopoietic cells but rather to depletion of gut microbiota by antibiotic treatment. Along the same lines, it is possible that cytopenias in patients with CLDs, in part, could be attributed to fecal dysbiosis. Further experimental studies are warranted to investigate this hypothesis and whether fecal microbiota transplantation may prove beneficial to improving cytopenias in CLD patients.

## Figures and Tables

**Table 1:** Baseline characteristics of patients with CLD on antimicrobials compared with those not on antimicrobials (Total *n* = 196).

Parameter	Total (*n* = 196)	Patients with CLD on antimicrobials (*n* = 115)	Patients with CLD without antimicrobial therapy (*n* = 81)	*p* value
Age, years (mean ± SD)	46 ± 15	44 ± 18	49 ± 11	0.105

Gender: male, *n* (%)	157 (80%)	91 (79.1%)	66 (81.5%)	0.687

Etiology of CLD: *n* (%)				
(i) Ethanol	62 (31.6%)	34 (29.6%)	28 (34.6%)	0.235
(ii) NASH	42 (21.4%)	22 (19.1%)	20 (24.7%)	
(iii) Viral	9 (4.6%)	4 (3.5%)	5 (6.2%)	
(iv) Cryptogenic	41 (20.9%)	30 (26.1%)	11 (3.6%)	
(v) AIH	5 (2.6%)	4 (3.5%)	1 (1.2%)	
(vi) Vascular	20 (10.2%)	8 (7.0%)	12 (14.8%)	
(vii) NCPF	7 (3.6%)	5 (4.3%)	2 (2.5%)	
(viii) Others	10 (5%)	8 (7%)	2 (2.5%)	

Hb (g/dL)	9.5 (7.6–11.3)	8.8 (7.2–10.4)	10.5 (8.6–12.1)	**<0.001**

TLC (×10^9^/L)	5.0 (3.2–8.7)	2.8 (2.5–5.6)	5.0 (3.1–9.1)	**0.048**

Plt count (×10^9^/L)	86 (49–154)	86 (43–135)	90 (54–165)	**0.043**

MELD score	11 (7.0–17.3)	13 (6.5–20.7)	12 (8.0–13.7)	0.201

Spleen size	15.7 ± 4.3	15.3 ± 4.0	16.4 ± 4.5	0.075

Procalcitonin	1.5 (0.3–1.2)	2.5 (1.3–2.7)	0.5 (0.3–0.5)	**0.011**

C-reactive protein	20.8 (7.9–37.1)	17.6 (7.5–29.3)	4.3 (3.9–10.9)	**0.035**

Ferritin	179 (76–592)	336 (79–691)	245 (70–544)	0.401

TNF-a (*n* = 16)	18.4 (17.3–29.4)	19.6 (17.5–32.9)	10.2 (8.2–29.4)	0.143

Antimicrobials used: *n* (%)				
(i) Cephalosporin	86 (44%)	86 (74.7%)	-	-
(ii) Carbapenem	40 (20.4%)	40 (34.8%)		
(iii) Beta lactams	25 (12.8%)	25 (21.7%)		
(iv) Quinolones	13 (6.6%)	13 (11.3%)		
(v) Antifungals	19 (9.7%)	19 (16.5%)		

Bone marrow impression: *n* (%)				
(i) Reactive	168 (85.7%)	89 (77.4%)	79 (97.5%)	**<0.001**
(ii) Hypocellular	25 (12.8%)	25 (21.7%)	0	
(iii) Inadequate (diluted marrow)	3 (1.5%)	1 (8.7%)	2 (2.5%)	

Outcome: *n* (%)				
(i) Discharged	169 (86.2%)	99 (86.1%)	70 (86.4%)	**0.081**
(ii) Dead	13 (6.6%)	15 (13.9%)	2 (2.5%)	**0.093**
(iii) LAMA	14 (7.1%)	1 (8.7%)	13 (16%)	

**Table 2:** The range of cytopenias and outcomes in different categories of antimicrobials in patients with CLD (Total *n* = 196).

Item	No antimicrobials (*n* = 81)	Cephalosporin (*n* = 86)	Carbapenem (*n* = 40)	Beta-lactams (*n* = 25)	Quinolones (*n* = 13)	Antifungals (*n* = 19)
Hb (g/dL)	10.5 (8.6–12)	8.8 (7.2–10)	9 (6.7–10.8)	8.8 (7.6–9.9)	8.6 (6.5–10)	8 (6.7–10.8)
TLC (×10^9^/L)	5.0 (3.5–7.6)	4.9 (3.1–8.7)	6.6 (3.3–12)	5.1 (2.5–11)	8.7 (2.4–14)	4.4 (3.1–12)
Plt count (×10^9^/L)	90 (54–165)	84.5 (40–154)	90 (51–150)	65 (41–110)	64 (23–104)	90 (50–176)
MELD score	12 (8.0–13.7)	11.5 (5.5–19.4)	18.5 (11–28.6)	15.4 (7.6–21)	15.6 (7.4–32)	12.7 (5–31.4)
Outcome, alive: *n* (%)	70 (86.4%)	75 (87.2%)	21 (52.5%)	19 (76%)	9 (69.2%)	10 (53%)

**Table 3:** Cytopenias among those who received antimicrobials and those who did not (Total *n* = 196).

Antimicrobial agent	Parameter	Those who received	Those who did not receive	OR (95% CI)	*p* value
Cephalosporins (*n* = 86)	Anemia	53.5%	38.2%	1.91 (0.073–3.409)	0.128
	Leucopenia	36%	33.6%	1.08 (0.597–1.957)	0.796
	Thrombocytopenia	58.1%	58.2%	1.00 (0.565–1.775)	0.995

Carbapenems (*n* = 40)	Anemia	50%	43.6%	1.294 (0.645–2.595)	0.468
	Leucopenia	27.5%	36.5%	0.659 (0.306–1.418)	0.286
	Thrombocytopenia	57.5%	58.3%	0.966 (0.478–1.952)	0.924

Beta-lactams (*n* = 25)	Anemia	56%	43.3%	1.667 (0.716–3.886)	0.236
	Leucopenia	40%	33.9%	1.299 (0.549–3.071)	0.551
	Thrombocytopenia	68%	36.7%	**1.621 (1.464–2.960)**	**0.041**

Quinolones (*n* = 13)	Anemia	53.8%	44.3%	1.469 (0.475–4.542)	0.504
	Leucopenia	30.8%	35%	0.826 (0.245–2.789)	0.759
	Thrombocytopenia	76.9%	46.8%	**1.532 (1.474–2.507)**	**0.039**

Antifungals (*n* = 19)	Anemia	57.9%	43.5%	1.786 (0.685–4.654)	0.236
	Leucopenia	31.6%	35%	0.856 (0.310–2.363)	0.764
	Thrombocytopenia	57.9%	58.2%	0.988 (0.379–2.576)	0.980
